# A qualitative study of the feasibility and community perception on the effectiveness of artemether-lumefantrine use in the context of home management of malaria in south-west Nigeria

**DOI:** 10.1186/1472-6963-8-119

**Published:** 2008-06-01

**Authors:** Ikeoluwapo O Ajayi, Catherine O Falade, Benjamin O Olley, Bidemi Yusuf, Sola Gbotosho, Toyin Iyiola, Omobola Olaniyan, Christian Happi, Kaendi Munguti, Franco Pagnoni

**Affiliations:** 1 Malaria Research Laboratories, Institute of Medical Research and Training, College of Medicine, University of Ibadan, Nigeria; 2 Department of Pharmacology & Therapeutics, College of Medicine, University of Medicine, University of Ibadan, Ibadan, Nigeria; 3 Ministry of Health, Oyo State, Nigeria; 4 Primary Health Care Unit, Ona-Ara Local Government, Oyo State, Nigeria; 5 Institute for Development Studies, College of Humanities and Social Studies, University of Nairobi, Nairobi, Kenya; 6 Implementation Research & Methods Unit, UNICEF/UNDP/World Bank/WHO Special Programme for Research and Training in Tropical Diseases (TDR), Geneva, Switzerland

## Abstract

**Background:**

In Nigeria ACT use at the community level has not been evaluated and the use of antimalarial drugs (commonly chloroquine (CQ)) at home has been shown to be largely incorrect. The treatment regimen of ACT is however more complicated than that of CQ. There is thus a need to determine the feasibility of using ACT at the home level and determine community perception on its use.

**Methods:**

A before and after qualitative study using key informant interviews (KII) and focus group discussions (FGDs) was conducted in selected villages in Ona-Ara local government area. At baseline, 14 FGDs and 14 KIIs were conducted. Thereafter, community medicine distributors (CMDs) were trained in each village to dispense artemeter-lumenfantrine (AL) to febrile children aged 6–59 months presumed to have uncomplicated malaria. After one year of drug distribution, nine KIIs and 10 FGDs were conducted. Participants and key informants were mothers and fathers with children under five years, traditional heads of communities, opinion leaders and health workers.

**Results:**

None of the participants have heard of AL prior to study. Participants were favourably disposed to introduction of AL into the community. Mothers/caregivers were said to have used AL in place of the orthodox drugs and herbs reported commonly used prior to study after commencement of AL distribution. The use of CMDs for drug distribution was acceptable to the participants and they were judged to be efficient as they were readily available, distributed correct dose of AL and mobilised the community effectively. AL was perceived to be very effective and no significant adverse event was reported. Major concerns to the sustainability of the program were the negative attitudes of health workers towards discharge of their duties, support to the CMDs and the need to provide CMDs incentives. In addition regular supply of drugs and adequate supervision of CMDs were advised.

**Conclusion:**

Our findings showed that the use of AL at home and community level is feasible with adequate training of community medicine distributors and caregivers. Community members perceived AL to be effective thus fostering acceptability. The negative attitudes of the health workers and issue of incentives to CMDs need to be addressed for successful scaling-up of ACT use at community level.

## Background

In Nigeria, malaria accounts for 25% of infant mortality and represents 8–12% of deaths in children less than 5 years [1]. The infection is commonly managed at home using orthodox and herbal medicines. When orthodox treatments are given at home, dosages are often incorrect and inadequate [2-4]. This may have contributed to the emergence of parasites resistant to the hitherto effective and cheap antimalarial drugs such as chloroquine and more recently sulphadoxine-pyrimethamine [5-8]

In an effort to stem the worsening morbidity and mortality due to drug resistant malaria, Nigeria changed its malaria treatment policy from chloroquine (CQ) or sulfadoxine-pyrimethamine (SP) to artemisinin based combination therapy (ACT) in line with the WHO recommendation [9], with a preference for artemether-lumefantrine (AL) in January 2005 [8]. In addition to the change of drug of first choice, the Federal Ministry of Health (FMOH) proposes to deploy ACT at community level in the context of home management of malaria (HMM). The dosage regimen for AL is however more complicated than that of CQ or SP. This underscores the need to determine the feasibility of using AL at the community level, ensuring adherence to dose regimen. In addition, there is a need to explore from the community perspective the effectiveness and safety of the drug when dispensed by non-medical personnel. Effort during this study was directed at this important issue in order to safe-guard the clinical useful life of this newer and effective antimalarial drug combination.

## Methods

### Study site

This study was conducted in 40 communities in two rural health districts selected by random sampling from the eight health districts that make up Ona-Ara Local Government area (LGA), in south western Nigeria from July 2005 to January 2007. A well established collaboration exists between researchers in the College of Medicine, the State Ministry of Health and the health unit of the Local Government (LG) having carried out studies on malaria in the LGA in the past. At the time of commencing this study, ACT has not been deployed to the LGA health facilities by the Government and was not available in the private sector either. The study site has been described in detail by Ajayi et al. 2008 [10].

### Health services in Ona-ara Local Government Area

The health care system (HCS) in Nigeria consists of three levels namely: primary, secondary and tertiary levels. The Local Governments are generally responsible for primary health care (PHC). In Ona-Ara LGA there is a Primary Health Care (PHC) unit at the LG headquarters and the coordinator of the unit is usually a medical doctor who oversees the activities of the health facilities in the LGA.

Each of the health districts has a health centre and maternity centre. The health centres are manned by a team of community health workers headed by a registered nurse/midwife. Few traditional healers and birth attendants (TBAs) provide indigenous health care in the districts. However, majority of the caregivers seek care from patent medicine sellers (PMS) and itinerant drug hawkers [10]. The populations of the villages in the two districts vary from 50 to 8,000 and are spread over a vast area with poor transportation and road network; making it difficult for the government health structures to be easily accessible or effective.

### Study design

This qualitative study is a component of a quasi-experimental study to evaluate the feasibility, accessibility and safety of using ACT for home management of malaria. To conduct a situation analysis prior to distribution of AL for HMM, Focus Group Discussions (FGDs) and Key Informant Interviews (KIIs) were viewed appropriate [11]. These were used to explore caregivers' and community members' opinions on malaria as it affects children, the prevailing treatment seeking behaviour including practice of home management of malaria and the potential for change. The findings of the situation analysis were used in the planning of the intervention including mechanism for drug distribution. After one year of intervention during which AL was distributed by selected and trained community based medicine distributors (CMDs) in study communities, the communities' perception on the use of AL for HMM, strategies for its distribution and accessibility to the people as well as acceptability were explored. In addition, household survey was conducted (to be reported separately). CMDs in this study comprised patent medicine sellers, health workers and mothers selected by the communities to be trained as "mother trainers".

### Sample population

Communities were selected for FGD and KII by random sampling technique from the 40 communities at baseline and after intervention respectively. The participants for the FGD which comprise mothers and fathers were selected using purposive sampling based on the recommendation by village head or opinion leader who perceived them to have sufficient knowledge and experience on the issues to be addressed and would be able to discuss freely [12]. In addition they must have a child less than five years or must have taken care of a child in the past. Focus group discussions (FGDs) were held in groups of six to nine women or men. The key informants were purposively selected based on their position in the community, their role in implementation, or because of the special information they possessed based on their involvement in the management of childhood fevers.

At baseline, a total of 14 FGDs comprising four groups of young mothers [aged <35 years], old mothers [≥ 35 yrs], and three groups each of young fathers [<35 years] and Old fathers [≥ 35 years] were conducted. Fourteen KIIs with six opinion/community leaders, four drug sellers and four health workers were also conducted. Post intervention, a total of 10 FGDs and nine KIIs were conducted. Three groups of young mothers, three of old mothers, two of young fathers and two groups of old fathers were involved in the FGD while three patent medicine sellers (PMS), four opinion/community leaders, and two health workers were interviewed during the KIIs. The total numbers of participants at the FGD sessions at baseline and post intervention were 103 and 89 respectively.

#### Conduct of qualitative study [FGD and KII]

The interviews were conducted by trained research assistants. Two teams of three assistants (moderator, note keeper and recorder) conducted the sessions. The teams were supervised by the social scientist (BO) on the research team. The research assistants had a 4-day training prior to conduct of the study. Training included familiarization with research tools and practicing interviews. Research assistants were also taught basic techniques of probing, recording responses, and note-taking as well as transcription of recordings. Fundamentals of organizing and conducting FGDs were also included in the training. Field guides were developed and pre-tested prior to use. The guides were translated into Yoruba – the local language.

Participants at both the FGDs and KIIs were shown the tape recorder and verbal informed consent to carry out the exercise and to record the sessions and interviews was obtained from each participant. The FGD sessions and KII lasted between 40–80 minutes. Interviews and FGDs were conducted to the point at which no new information was forthcoming. The participants were given a pack containing multivitamins and acetaminophen (paracetamol) at the end of each session. Transcription was completed daily.

### Intervention Activities

#### Selection of CMDs

Intensive advocacy and sensitization of community heads, opinion leaders and residents of the selected communities were carried out in the company of the LG PHC unit coordinator and malaria control program officers. The community heads were briefed about the purpose of the study and method of collection of information. They were requested to select mothers to be trained as "mother trainers" from their communities. Selection criteria for CMDs included being a permanent resident (at least one year), trusted and respected by the community, able to keep simple records, and a willingness to serve. In addition, the selected mothers should have the consent of their husbands. Selected mothers and the existing patent medicine sellers and itinerant drug hawkers were trained and they served as community based medicine distributors.

#### Treatment guideline

**Figure 1:**
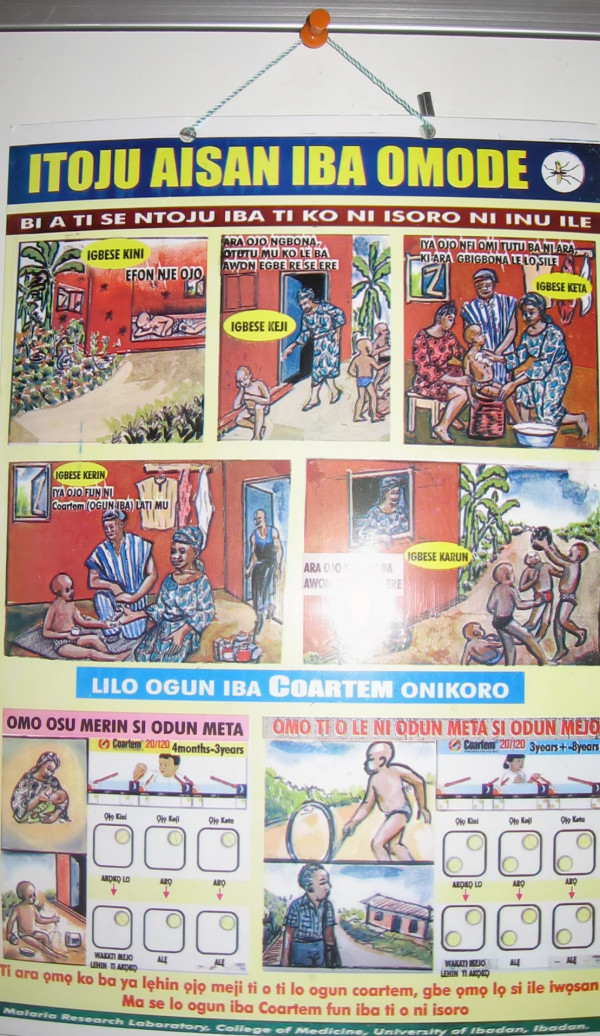
Treatment Guideline depicting some symptoms of uncomplicated malaria and the dosage regimen for Coartem^®^.

A treatment guideline developed for a past study on HMM using chloroquine [10] was adapted for ACT use in this study and pre-tested prior to distribution to every household in the study communities by the CMDs. The guideline was in cartoon format and had pictorial illustrations of some common clinical features of uncomplicated malaria, the correct dose and regimen of AL according to the age of the child [Fig 1]. Training was conducted for caregivers by the CMDs in their communities to improve their ability to recognise uncomplicated and severe malaria and on how to use the guideline.

#### Study Drug and distribution

The drug of choice for this study was AL (Coartem^®^; Novartis) in line with the FMOH choice of ACT. The recommended dose for children aged six to 35 months was one tablet twice daily for three days, and for children aged 36 to 59 months two tablets twice daily for three days. Community medicine distributors (CMDs) were trained to dispense AL to caregivers of febrile children aged 6–59 months presenting to them, after exclusion of danger signs. They provided instructions on dose regimen, possible adverse event and danger signs. Caregivers were advised to administer the drug with or after meals, preferably fatty food. The caregivers then administer the drug to their children at home unsupervised. Direct observation or active follow-up of caregivers by CMDs was not recommended. Monthly supervision was carried out by research staff to check entries in CMDs record and drug stocks. The supply of AL was from Global Fund to fight Malaria/TB/HIV/AIDs and was distributed through the FMOH, Nigeria. Drugs were provided through the existing health centres in the districts. CMDs replenished their stock at monthly meetings at the health facilities or as the need arose. In some instances drugs were delivered by the research staff during supervisory visits.

In the spirit of community participation and ownership, the team and community decided that the drug should be sold at an affordable price to the caregivers. At focus group meetings with CMDs and community members prices which ranged from ₦10.00 to ₦250.00 (8 cents to 2 USD) were suggested to be charged for AL. However, ₦20.00 (16 cents) was mentioned to be the most affordable for caregivers. Thereafter, having made due consultation with community heads and opinion leaders, it was agreed that the 6 × 1 dose pack be sold for ₦30.00 (24 cents) and the 2 × 6 dose pack for ₦50.00 (40 cents). The team and CMDs agreed that a commission of ₦30.00 (24 cents) on every pack of AL sold should be given as incentive to cover the profit the PMS would have made normally on selling other antimalarial drugs as well as to compensate for the time the CMDs were likely to spend attending to a febrile child. However, this had to be revised at six months into the distribution of AL in line with the FMOH policy that it should be made free to children. The project had to take up payment of the incentives as agreed upon in other to motivate the CMDs and to ensure smooth continuation of the study.

### Analysis

All interviews were tape recorded and transcribed in Yoruba the language which was used to conduct interviews and FGDs. Two of the research assistants and the social scientist (BO) and one of the authors (IOA) listened to the recordings, checked the accuracy of the transcripts and translated the transcript from Yoruba to English. Content analysis was performed independently by two of the authors IOA and BO the social scientist. They compared notes for congruency and where there was incongruence they re-read the transcript and made necessary corrections From the transcripts and field notes, the responses from different informants or groups (FGD) to questions asked to explore issues of interest were grouped together, coded and analyzed according to themes related to recognition of malaria, treatment practices, use and adherence to use of AL, adherence of CMDs, effectiveness and safety of AL, drug distribution mechanism and sustainability of the intervention. The findings were described, interpreted and reported in form of narratives.

### Ethics

Ethical approval for the study was provided at national level by the Oyo State Ethical Review Board and the Ethics Review Committee of the WHO. Informed consent was obtained from community heads, household heads and the caregivers who participated in the study.

## Results

The results are presented in two parts. The first part is the exploration of the knowledge of caregivers about malaria, the prevailing treatment seeking behaviour in the community prior to the intervention and the participants' involvement in determining the intervention activities. The second part reported findings at assessment of intervention one year after distribution of AL.

### Knowledge and Recognition of Malaria in Children

An important exploratory study was conducted at the beginning of the FGDs to ascertain the study communities' definition and nomenclature for malaria. The locally recognized syndromes in children treated with antimalarial drugs by participants and the local terminology *'iba' *were explored. Most of the FGD participants described *"iba"*- the local terminology for fever as high temperature associated with chills/rigors, vomiting, anorexia, headache and weakness. This definition is consistent with the symptomatology of malaria as seen in clinical practice. The FGD participants also mentioned that *"iba" *when literarily translated means fever, *"iba" *is as such also used for other febrile illnesses not presumed to be malaria. However, if a febrile illness is perceived to be from another cause the *"iba" *terminology is further qualified. Examples given by FGD participants are: a febrile illness perceived to be caused by measles, is called *"iba ogbele". "Ogbele" *is the Yoruba word for measles and *"iba typhoid" *if the fever is perceived to be due to typhoid fever. This preliminary finding was useful in ensuring that researchers and community members were in agreement in the use of the terminology for malaria.

Malaria in children was perceived to be a serious problem in the community and the general consensus among the participants was that it kills children fast if care is not sought promptly. A young mother said: "*malaria is a major health problem because if it stays too long in a child's body, it drains the blood"*.

The participants demonstrated good knowledge of the symptoms and signs of malaria. The symptoms and signs participants associated with uncomplicated malaria included fever, chills, excessive weakness, anorexia, vomiting, excessive sleep, 'stretching of the body', concentrated urine, headache and dull looking eyes. For severe malaria, they mentioned very high fever, convulsion/fits, restlessness, prostration, excessive vomiting and weakness, yellow eyes and dark coloured urine. Some respondents mentioned they suspect severe malaria when a febrile child is not responding to the treatment first given.

#### Treatment practices

The prevailing treatment practice for malaria in children at onset of this study was the use of orthodox drugs bought from patent medicine sellers (PMS) or drug hawkers and the use of local herbs at home. The option first explored depended on availability. Commonly, herbs were mentioned to be readily available hence are used first in many instances. This is usually followed by orthodox drugs if available soon after in addition to the herbs already given. In situations that a caregiver has orthodox drug at home this may be given first. Treatment was reported to be instituted promptly mostly within 24 hrs of noticing fever by all participants.

It is only when the herbs or drugs used are found not to be effective that child is taken to the health care facility. Referral often takes place after about 2–3 days of treatment at home. During one of the FGDs a young mother said: *"the first thing we do is to prepare herbs for the child when the body is hot and administer it before drug hawkers come because they don't come regularly"*.

This was corroborated by health workers at KII. One reported thus: *"many of the parents first treat their children with herbs. They may also buy drugs from patent medicine sellers and combine with herbs. The reason why they buy drugs from hawkers is that they think the method will work; they come to the health centre when the illness is not responding to the drugs already used"*.

FGD participants also mentioned that children considered to have severe malaria were usually first treated with herbs and then taken to the health centre when there is no improvement after administration of herbal preparations.

A young mother said: "*we give herbs and use leaf and palm oil or black soap to wash the child so that child will get better before taking the child to anywhere (health facility or patent medicine seller)"*.

The orthodox drugs commonly used in the home management of malaria were chloroquine and paracetamol. However, many mentioned that they lacked adequate knowledge of the correct use of these drugs and relied on drug sellers' instructions. The common sources of drugs in the communities were itinerant drug hawkers and patent medicine sellers who sometimes also hawk drugs. The major barriers to accessing health facilities identified were non-availability or erratic supply of drugs in the health care facilities, distance of some of the communities to the health centres, poor transportation system in the rural areas and financial constraints on the part of the caregivers. Other barriers were related to the negative attitudinal disposition of the health workers. These include absenteeism of health workers [HW] and unauthorized charges for treatment, which may not be affordable. Reasons proffered for HW misdemeanour include HWs' displeasure at being posted to the rural area and problems with transportation from the city [where most of them reside] to the health centre in the rural area; while some participants believed that some of the HWs were just out to make money.

The estimated cost of treatment (based on caregivers' judgment) for an episode of febrile illness in a child if orthodox drugs were used ranged from ₦100.00 – ₦7000.00 [90 cents to 55 USD). The estimated cost for home management ranged from ₦100 – ₦500 [90 cents – 4 USD] while treatment at health facilities was estimated at ₦500 – ₦2, 000.00 [4 USD – 17 USD] for out patient treatment and ₦2,000.00 – ₦7,000.00 [17 USD – 55 USD] if the child was admitted for in-patient care. These estimated costs included cost of the different types of drugs given and the cost of transportation which ranged from ₦20.00 to ₦500 [16 cents – 4 USD] to travel to nearby or distant village or to the city of Ibadan.

#### Awareness and willingness to use artemether-lumefantrine (AL)

None of the participants at FGD had heard of AL at baseline. A few of the health workers mentioned they had learned of government's intention to distribute the drug but have not seen it before. All the participants were favourably disposed to the introduction of AL for treatment of malaria in their children provided it is an effective drug. One young mother said:

*"Since you say it is very effective, bring it for our children. We will have peace of mind because the incidence of children falling sick often and often will be reduced"*.

The older mothers and fathers referred to the caretaker role of the government. They mentioned that it is the responsibility of the government to look after the health of her people, as such; *"it is a good idea, if the government introduces it into the community"*.

#### Comprehension and Understanding of the Package of AL

**Figure 2:**
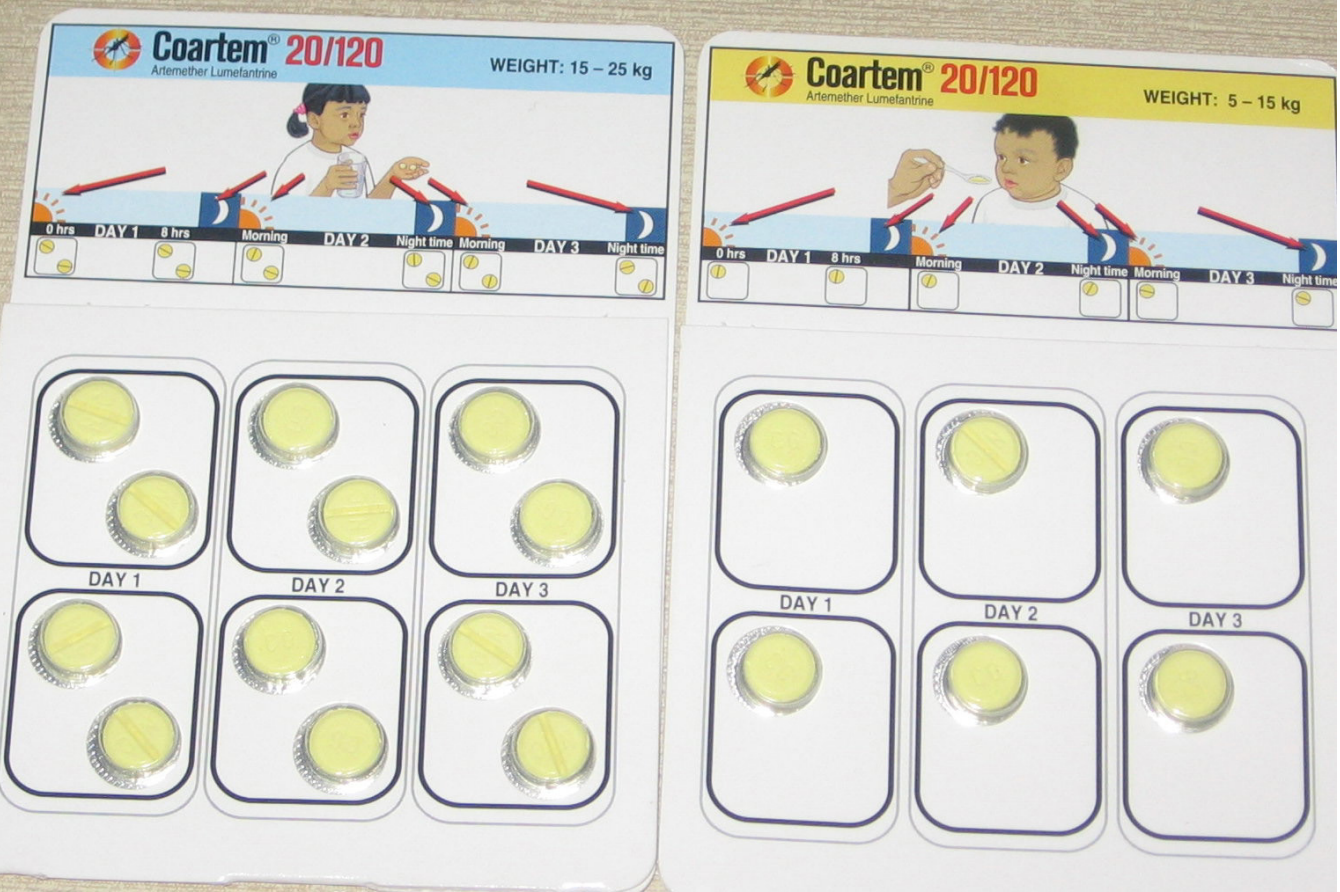
Picture showing the packs of Coartem^® ^Used [2 × 6 & 1 × 6].

In order to determine the FGD and KII participants' understanding of the illustrations on the package of AL and the appropriateness to the local setting, samples of the AL (Fig 2) to be distributed were shown to the participants at baseline. Most of the participants recognized the pack of AL as drug meant to be taken by children because of the picture of a child on the pack. However, their explanations on the use based on illustrations on the pack were mostly incorrect. Some said it is for malaria because the colour of the tablet is yellow and because a mosquito which was perceived to be a cause of malaria is drawn on the pack. Many participants could not comprehend how to administer the drug by just studying the drawing on the pack. The responses are that one tablet of AL is to be used three times a day for two days (1 × 6 pack); one daily for six days (1 × 6 pack) or twelve days (2 × 6 pack) and that the tablet is to be divided into two because each tablet has a line down its middle.

They requested to be given verbal instruction and training on how to use the drugs for better understanding and correct usage.

#### Opinion about drug distribution process

There were mixed opinions amongst participants regarding the preferred outlet for the distribution of AL. The outlets mentioned were the existing health centres, drug sellers (PMS and drug hawkers), trained mothers, community leaders and research team. The most mentioned was through trained mothers. A few mentioned that drug should be distributed to each household based on the number of children therein. However, from the reasons given for preferring one to the other, many of the respondents raised objections to the use of drug sellers especially drug hawkers followed by health workers. Only a few raised objections to use of mothers while none raised objection to the use of community leaders and research team. The FGD participants were of the opinion that the PMS and health workers will sell the drugs to them even if meant to be distributed free or hike the price if they were to be sold. The anxiety expressed by the mothers and caregivers stemmed from past experiences where by some drug sellers and health workers had demanded money for drugs that were meant to be free or hoard the drugs for their own private use. Another concern expressed by the mothers was the HWs irregular attendance at work. Quotes to support these responses included:

*"Yes, use mothers for the distribution of the drug in this community, don't use drug hawkers, because they may sell it and people that cannot afford it will not be able to buy "*- said a young father.

A "traditional ruler" of one of the communities said: "*Give the drug to us, we will give it to the mothers and also give it to health workers at the health centres to give to children that are brought to the centre"*.

### Assessment of intervention after one year of distributing AL

#### Awareness and use of AL

Most of the participants at FGDs held after one year of distributing AL mentioned that they have heard about AL. A few participants in the FGD groups for fathers mentioned that they have not heard about the drug. One quotation to corroborate their awareness goes thus:

*Yes, we have heard about the drug and have the treatment guidelines. It is for treatment of malaria in children and it is good" *said *a *young mother. Participants also mentioned that they used AL to treat their children during episodes of febrile illness presumed to be malaria. This was supported by this quotation:*"since you introduced the drug into our community, mothers go to the CMDs to collect drug for febrile children presumed to have iba"*. Treatment was reported to be instituted promptly mostly within 24 hrs of noticing fever by all participants. A young father said:

*"Mothers go to collect drugs from CMDs as soon as they notice "iba" in a child. They use the drug quickly, because they know if they delay, the illness may progress and prevent them from going to their work"*.

Most of the participants mentioned that children suffering from severe malaria are supposed to be taken to the hospital/health centre as the first line of treatment. However, few participants, mostly older mothers' and older fathers' still mentioned herbs as first line treatment and would only take such children to hospital if there was no improvement. In one FGD group, a mother admitted that she took her child to the health centre after tepid sponging and putting a mouth gag (teaspoon wrapped with cloth) when her child had symptoms suggestive of severe malaria (very high fever, vomiting and convulsion/fits).

Most of the participants who had used AL mentioned they were able to understand the dosing schedule after the training by CMDs who also provided instructions at the point of collection of the drug. The participants also mentioned that the guideline which was provided as hand bills to the households aided their understanding of the dosage regimen for AL. However, a few FGD participants who were mainly fathers and "old mothers" mentioned they did not understand the illustrations on the pack. All the participants who had used AL mentioned that the cost of treatment of a febrile child reduced drastically with the distribution of AL. The cost was less than 50 cents (USD) when AL was being sold. When it became free, the cost incurred was mainly for other drugs such as paracetamol and multivitamins when prescribed. There was no transportation cost since drug was available within walking distance.

#### Drug Distribution

At the end of the intervention, participants expressed satisfaction with the idea of keeping drugs with people within the community. They mentioned that PMS and "mother trainers" were not far from them and were always available when needed be it day or night.

One young man attested to the effectiveness of the 'mother trainer' in his community. He said: *"The mother trainer you selected in my community put a lot of effort to ensure that people know about the drug and use it. She goes around educating people, creating awareness and she is always available. Please help to reward her"*.

None of the participants mentioned that any of the CMDs charged illegal fees. Many of the young fathers mentioned the drug was given to the caregivers free. During KII, opinion leaders mentioned that they appreciated the wide distribution and availability of the drugs within the community. They also mentioned that people in the neighbouring communities took advantage of the facility and that drugs were always available unless the CMD was out of stock. A young mother said: *CMDs are always available. It is good we have two in our community, when one is out of the village the other one attends to caregivers*. One participant expressed the view that it would be useful if CMDs are allowed to hawk AL because many people in the rural areas cannot afford the cost of transportation.

Patent medicine sellers and health workers welcomed the involvement of PMS and trained mothers for distributing AL. This view was premised on the fact that PMS and community members were readily available compared to health workers who are available only during official hours and sometimes irregularly. PMS had some reservations about having to replenish their stock at the health centres. The PMS would rather have drug stocks supplied to them directly by the research team. They were also willing to collect AL directly from the drug store at LG headquarters. The PMS were dissatisfied because they have had to make repeated visits to health centres before they could replenish their stock as the health workers were either not at their posts or they have closed before CMDs get to the health centre. One said: "*some of us do not have time to go to health centre especially when repeated visits have to be made"*.

#### Adherence to use of AL and occurrence of adverse event

The consensus during the FGDs and KIIs conducted post intervention was that the people in the community used AL very well. A community head said: "*all children in my community used it whenever they have a febrile illness. The drug is very good and we will like the use extended to older members of the community too"*.

Participants at FGDs comprising mostly young mothers and fathers mentioned that they refer to the treatment guideline to check for the dose of AL. Most of the FGD participants mentioned that the mothers completed the dose as instructed and this was corroborated by PMS and health workers. However, some participants in the young fathers' group mentioned that not all the mothers completed the dose of AL. One of the participants specifically mentioned that his wife did not complete the dose in the treatment of their child. When the issue of non-completion of AL dosage regimen was discussed further, the fathers mentioned that some mothers stopped administration of AL when the children are better. Some mothers however disagreed and maintained that non-completion of dosage regimen was an exception. One young woman corroborating the reason given by the young fathers said: "*Some mothers may forget to complete the dose more so when their children get better and are playing around"*.

#### Effectiveness and Safety of AL

All the participants mentioned that AL was very effective and that they were satisfied with it. A PMS said: "*The child I administered the drug to got well and started playing soon after commencement of the drug; the drug worked very well beyond our expectations"*.

An old mother was particularly impressed with the rapid improvement observed in the health of the ill children following administration of the AL. She said "*whenever a child is given the drug, the fever goes down quickly; child starts to eat better and goes to play by the next day. It makes the children strong again"*. A young father corroborated the assertion and said: "*the use of AL has led to a marked reduction in time caregivers spend with a sick child because the children now recover promptly and by the second day of use many of the sick children were up and about"*.

Reports of adverse events following the use of AL were few and far between. When asked about adverse events, one woman noticed skin rash which she attributed to measles infection. One PMS mentioned that he heard that a few of the children in another community experienced mild skin rash and passing of dark stool. However, these were not serious and did not warrant an intervention.

#### Opinion about the implementation of the program

The consensus among all participants was that the program went well and they perceived AL to be effective in the treatment of malaria in children. An opinion leader said:

*the project has been very useful to us as a community and our children. Since the distribution of AL started, our children have been receiving prompt and effective treatment for malaria. All those treated with it got well with no adverse event. The drug is good*.

The health workers stated particularly that they pray that the collaboration between the health centre and the other CMDs continues.

#### Sustainability of the programme in the community

The issue of sustainability of the programme was a major concern. Before winding up the study, meetings were held with stake holders in the two health districts to deliberate on possible ways of sustaining the program when the research team must have withdrawn their services. A number of suggestions were made at FGDs with caregivers and KIIs with PMS and opinion leaders. All the suggestions had the desire for sustainability of the programme in common. Other key suggestions concerning sustainability of the programme were:

▪ It was suggested that community leaders make it mandatory for community members to donate towards a fund which is to be set up with the sole aim of providing transport or transport fares to CMDs to collect drug from distribution point and providing incentives to the CMDs.

▪ The community should cooperate with the CMDs and give them the required support. The participants mentioned that they will be ready to assist CMDs create awareness.

▪ Some FGD participants mentioned that the Government should adopt the programme and continue its implementation

▪ FGD participants were particularly insistent that CMDs be remunerated as this will serve as motivation to continue their laudable service to the community. The remuneration was considered necessary because CMDs were sacrificing time they should spend pursuing their personal business on community service.

▪ Drugs should be made available at all times and should be stored only by trained CMDs. It was also suggested that drugs should be supplied directly to the PMS and 'mother trainers' rather than going to health centre to replenish stock. By so doing the community will be able to monitor the distribution of the drug better, said a PMS.

▪ Research team or LG representative should pay supervisory visits to the CMDs and communities.

## Discussion

Home treatment of malaria using both orthodox medicine and local herbal remedies is a common practice in the study communities as well as many other rural communities in Nigeria [13]. The low utilization of health facilities as the first resort for malaria treatment in this study corroborates reports from other African countries [13,14]. Poor access to health care services and orthodox drugs in rural areas as a result of their remote location and poor socio-economic status is well recognized and constitute an issue of inequality of access when compared to caregivers in the cities and urban area. This and the fact that incorrect use of orthodox drugs is common underscore the need to determine the feasibility of introducing a new antimalarial drug to the community especially rural and to assess the community perception on its effectiveness.

### Acceptability

The caregivers, CMDs and other members of the communities in which this study was conducted were very receptive to the new drug (AL). The easy and convenient access of community members to AL at reasonable cost initially and latter at no cost and the observed rapid improvement in the state of health of children treated with AL must have contributed in no small measure to the acceptability by the community. The fact that the AL distributed was pre-packaged could also have contributed to the acceptability. The acceptability of the blister pack is consistent with results from a study in western Uganda [15] and rural communities in Nigeria [13]. These studies found that caregivers and drug distributors preferred pre-packed drugs over loosely packed tablets. The mentioned reasons for this were safety, cleanliness and possibly facilitate correct dosing. To further demonstrate the acceptability of AL is the demand for extension of the intervention to the treatment of malaria in adults and neighbouring communities. Community members were also willing to support any measure that could ensure continued availability of AL in their communities.

The superior efficacy of AL in the management of malaria when compared to that of chloroquine which hitherto was the antimalarial drug of choice has been well established in clinical studies [8,16,17]. This study can only mention that caregivers perceived AL to be effective but cannot draw an objective conclusion on its effectiveness because it is a small scale study based on purposive sampling. However, the effectiveness of AL when distributed at the community level by community members was tested in an effectiveness study nested into this study (to be reported in another paper).

### Drug adherence and safety

Two main concerns in introducing ACT for use in HMM context were adherence and safety of use. These were borne out of the fact that the effectiveness and safety of AL at the community level have not been evaluated in Nigeria. In addition, the practice of HMM using orthodox medicine has been shown to be largely incorrect in Nigeria [2,13,18]. Some of the challenges identified in past interventions carried out to improve HMM were also identified in this study. These include failure of caregivers to complete a full course of antimalarial drug including AL [2,19,20], provision of financial motivation to CMDs [21] and non-adherence of health workers to recommendations on the use of AL [22].

In this study, adherence to correct dose of AL by caregivers was attested to by participants at FGDs and KIIs. Although this was subjective and from the perspective of the consumers, there is a likelihood that this was the case as some caregivers who used the drug were able to correctly recall how the drug was used when asked by CMDs. This finding is reassuring in view of potential to development of resistance. However, the perception of some participants in this study that some caregivers sometimes genuinely forget to continue therapy once the ill child appears well or deliberately keep the remaining drug for latter use for another child who falls ill should not be overlooked. Unknown to caregivers, parasite clearance is not necessarily complete before the patient improves. This practice if uncorrected will encourage emergence of drug resistant parasites as was witnessed with chloroquine. There is thus the need to provide health education to the public and healthcare workers alike emphasizing the need to always complete the treatment regimen of not just AL but all ACTs.

Prompt treatment of malaria is an important factor in the prognosis of the illness. It is encouraging that caregivers in this study were said to have commenced treatment promptly. This finding is similar to reports in other past studies in the study environment [13,18]. This attitude is favourable to implementation of home management of malaria strategy which emphasizes prompt and correct treatment. The malaria control unit of the Federal Ministry of Health, Nigeria should build upon this attribute of the caregivers in the introduction of AL into the community.

Throughout the study period, AL was perceived to be safe with no report of significant adverse event by the communities. Although this finding is similar to reports of earlier studies which reported mild adverse effect during clinical trials [16,17] and among patients prescribed AL [22], it cannot be used to draw conclusion on the safety of AL because of the small scale nature of the study and the qualitative design both of which constitute a limitation to the generalizability of findings in this study. Despite this seemingly good safety, the Federal Government and all stake holders should put an effective pharmaco-vigilance system in place when AL is deployed on a large scale.

Many interventions to improve adherence to correct dose of antimalarial drug at home level have been demonstrated to be effective [14,23-26] and this study is not an exception. However, sustainability of the correct practice is a major concern. This was addressed in this study by providing a pictorial guide which was comprehensible to the rural dwellers and served as a reference document even after the researchers might have moved out of the community. The availability of a trained resource person within the community who they can contact in case of doubt is another modality put in place to address the problem. Although these modalities appeared to have helped in ensuring adherence during intervention, there is a need to assess their effectiveness at regular intervals after the research team must have withdrawn from the communities.

### Drug distribution

The drug distribution mechanism proposed by community members varied at baseline. While many reasonably favoured the use of the drug sellers (PMS and drug hawkers) who were the main supplier of drugs in these areas, there were misgivings by a handful of other participants who would not want them involved because of distrust and the perceived propensity of the drug sellers to maximize profit. However, this attitude was not demonstrated by drug sellers during intervention in this study. On the contrary, caregivers perceived drug sellers to be efficient and accessible. There was no report of drug sellers charging illegal fees at any time during the study. The reported good adherence by the drug sellers was probably enhanced by the incentives by way of commission they received on each pack of AL sold or distributed and commitment to their community's health and development.

### CMDs' performance

"Mother trainers" and PMS were perceived by caregivers and other community members to be effective in this study. Not much was said about the efficiency of the health workers probably because they did not have to go to the health facility and consequently had minimal interaction with them. However, drug sellers and "mother trainers" who had to go to the health facility to replenish their drug stock had a lot of reservations about their effectiveness. They reported that health workers were often absent from their duty post and were perceived to hoard the study drug (AL). This attitude of the health workers made the CMDs to prefer drug distributed to them directly through other channels such as directly from the LGA drug store and work under the supervision of community leaders rather than health workers. The use of community members to assess efficiency of health workers and vice versa could be fraught with biases and prejudice existing prior to the study. This not withstanding the negative attitude of health workers if unchecked poses a major threat to the deployment of AL at the community level in Nigeria. Home management should not be seen as an alternative to strengthening health systems. The Primary Health care system needs to be strengthened and effective monitoring put in place in order to ensure a sustainable home management program. Following these a functioning linkage between the health facilities and the CMDs as well as community leaders should be fostered. This linkage is important to support HMM strategy because the health care facilities are needed for supervisory role and management of referred cases.

### Sustainability of program and CMDs' performance

This study has demonstrated that community volunteers and patent medicine sellers in the communities constitute an effective channel for intervention which is similar to findings by Marsh et al. [25]. The CMDs effective performance in this study was facilitated by the intervention in form of training provided to them prior to assuming their role as drug distributors and provision of incentives to sustain behaviour change [27]. However, the sustainability of their involvement and the regular supply of drug is a major concern. One suggestion to sustaining the involvement of the CMDs is maintenance of training and supervision of their activities. However, where sellers lack financial incentives to behave as they have been trained, they tend to revert back to inappropriate practices [27].

Incentives which may not be sustainable contributed significantly to the good performance of the CMDs during this study. Four strategies have been advanced for improving the practices of retail pharmacies in developing countries: information, persuasion, incentives and coercion [28]. The use of incentives to promote interventions and change in behaviour remains a controversial issue [21,29]. The outcome of this study suggests that if used appropriately incentives can positively affect performance. In the study by Wasunna et al. [22], health care workers at facility level demonstrated concern on the sustainability of a program that provides expensive drugs free to the people and the fear of drug being out-of-stock resulted in non-adherence by the health workers to distribute drug as recommended. In an effort to ensure stocks last, the health workers prescribed AL only to those they perceived to have serious illness. Could the perception that health workers hoarded drug in this study be related to the perceive fear exhibited by health workers in Wassuna et al. study but unspoken?

One way to address the issue of sustainability of community based programme is community partnership and participation strategy [30]. Efforts were made to address this during the study. The engagement of the CMDs including selected mothers was a demonstration of community participation. However, more could not be done due to the prevailing free drug policy by the government. A caveat to the use of this strategy is that organizers of most community based interventions seem to assume that because malaria is a major cause of mortality community members will volunteer their time free of charge to be trained, or train others, to act as health educators and drug distributors [19]. However, this has been shown to be fraught with problems. This was suggested in this study in which the CMDs suggested the need to provide commission to make up for the profit they would have made selling other antimalarial drug and time used in carrying out the assignment despite the fact that it was made known to them that it was a community volunteering work.

To scale up HMM using ACTs the government needs to consider these challenges seriously and addressed them in order to achieve successful implementation. In Nigeria, the private sector, which consists of a great variety of providers including CMDs, is used by a wide cross-section of the population [31]. Using the private sector to distribute drugs is a good option to address the challenges faced by consumers in the public sector. However, the practices of medicine sellers in low-income countries have been demonstrated to be fraught with many incorrect practices and these constitute a major challenge to working with them. Infringement of health-related regulation is extremely common [32]. Most stores lack permit, stock unregistered and/or expired drugs, stock prescription-only medicines, sell drugs at inflated prices and the staff do not have the minimum qualification required. The evidence is limited as to which approaches work best for public-private collaboration to improve medicine sellers-based treatment. Eliminating regulatory infringement is unlikely to be feasible and could be undesirable if access to essential medicines is reduced [32]. Alternatives include bringing official drug regulation closer into line with locally legitimate practices; greater use of positive incentives for providers; and consumer involvement [32]. In this study as mentioned earlier, training and provision of incentives were used and suggestions were made to the involvement of community heads or opinion leaders in supervising and monitoring drug distribution at the community level. However, there have been many references to other approaches such as social marketing, accreditation, franchising and contracting [33]. Further research is necessary to determine which of these strategies would be well suited for use in the context of HMM in Nigeria.

## Conclusion

The findings from this qualitative study showed that the use of AL at home and community level is feasible and acceptable to the members of the communities studied. AL was perceived to be very effective and safe. The use of CMDs made AL readily accessible and available in the community and the CMDs were found to be effective. These findings provide evidence to support scaling up implementation of HMM with ACTs in Nigeria. However, mechanisms to ensure long-term performance of CMDs, sustain provision of free drug and strengthen the health system should be developed.

## Competing interests

The authors declare that they have no competing interests.

## Authors' contributions

All the authors of this paper contributed to the conception and design of the study. BOO, IOA, BY and COF were instrumental in data collection and supervision. BOO and IOA undertook the analysis. TI and OO were instrumental to the approval of the study at the state and LG levels and facilitated advocacy and community mobilization. IOA produced the draft of the manuscripts and other authors reviewed and contributed to the final draft. All authors read and approved the final manuscript.

## Pre-publication history

The pre-publication history for this paper can be accessed here:


